# Potential use of olive oil mill wastewater for bacterial cellulose production

**DOI:** 10.1080/21655979.2022.2050492

**Published:** 2022-03-09

**Authors:** Taner Sar, Meltem Yesilcimen Akbas

**Affiliations:** a Swedish Centre for Resource Recovery, University of Borås, Borås, Sweden; b Department of Molecular Biology and Genetics, Gebze Technical University, Gebze-Kocaeli, Turkey

**Keywords:** Bacterial cellulose, bioconversion, olive oil processing, food waste, characterization

## Abstract

In this study, olive oil mill wastewater (OOMW), an important waste in the Mediterranean basin, was evaluated to produce bacterial cellulose (BC). For this purpose, the effects of different ratios of OOMW fractions (25–100%) and some additional nutrients (yeast extract, peptone and Hestrin-Schramm medium (HS) components) on BC productions were investigated. Unsupplemented OOMW medium (75% and 100%) yielded as much as BC obtained in HS medium (0.65 g/L), while enrichment of OOMW medium (%100) with yeast extract (5 g/L) and peptone (5 g/L) increased the amount of BC by 5.5 times, reaching to 5.33 g/L. In addition, produced BCs were characterized by FT-IR, TGA, XRD and SEM analyses. BC from OOMW medium (100% OOMW with supplementation) has a high thermal decomposition temperature (316.8°C), whereas it has lower crystallinity index (57%). According to the FT-IR analysis, it was observed that the components of OOMW might be absorbed by BCs. Thus, higher yield productions of BCs from OOMW media compared to BC obtained from HS medium indicate that olive oil industry wastes can be integrated into BC production for industrial applications.

## Graphical Abstract

**Figure:**
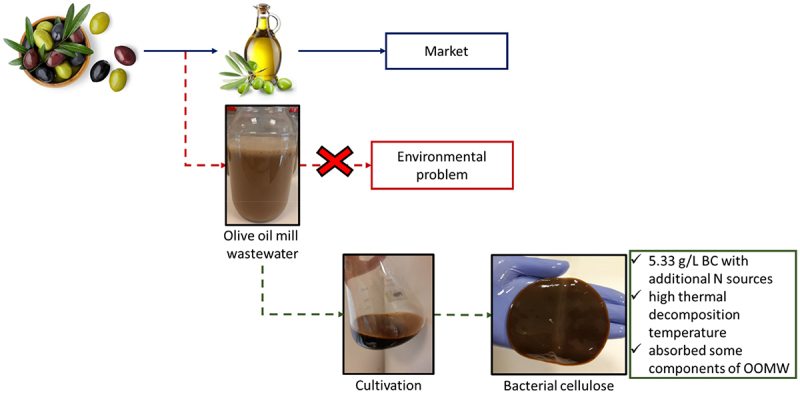

## 1. Introduction

Bacterial cellulose (BC) is a natural biomaterial that is produced by several microorganisms such as *Gluconacetobacter, Sarcina, Agrobacterium, Rhizobium, Rhodobacter*, and *Agrobacterium* [1,2]. Among them, *Gluconacetobacter* (known as *Acetobacter* and *Komagataeibacter*) which can be naturally isolated from Kombucha is the most common cellulose-producing microbial genera [^1–3^]. The structure of BC is similar to plant cellulose but does not contain lignin, hemicellulose and pectin [4]. It is also a popular biopolymer due to its mechanical stability, thermostability, high crystallinity (70–80%), high purity, low density, high specific surface area, excellent permeability, high porosity, high water content (up to 99%) and good biocompatibility [4–6]. Therefore, BC has been used as a biomaterial in food, paper, cosmetic, textile and energy industries [3,7–12]. Moreover, BC is known as generally recognized as a safe (GRAS) product and thus it can be safely used as stabilizing and gelling agents in the food industry [13,14].

BC production is extremely expensive due to the high cost of synthetic media used for its production [9,15]. To reduce the costs of BC production, several kinds of wastes as low-cost substrates (food industry, agricultural, brewery and beverages industries wastes waste) have been evaluated (reviewed in Hussain et al. [16]). Alternatively, olive oil mill wastewater (OOMW) can be a potential substrate for bacterial cellulose production. OOMW is a by-product abundantly released during traditional and commercial olive oil production, in Mediterranean basin [17]. OOMW, which is slightly acidic, contains sugars, lipids and aromatic compounds and has a high chemical oxygen demand [18]. OOMW has been used for the production of different biotechnological products such as enzymes, hydrogen, protein-rich microbial biomass, biosurfactants, etc., because of its rich content [17,19–22]. Pomace, another by-product of olive oil process, has also been investigated for productions of antioxidants (phenolic compounds) [23], biogas [24] and bacterial cellulose [25]. Since the pomace from the olive oil process can be used as a fertilizer [26] or animal feed [27], there is also a need to determine the advantages of another related waste, OOMW.

BC can be produced on different substrates including various fruit wastes [15,16]. Although pomace from olive oil processing was evaluated [25] to the best of our knowledge, there is no report for the use of OOMW for BC production. Therefore, in this study, it was aimed to investigate the production of BC from OOMW as a by-product of olive oil industry. For this, different fractions of OOMW (25%, 50%, 75% and 100%) and the effect of additional media components (supplementation of yeast extract, yeast extract and peptone, and HS medium components, separately) were investigated by comparing them with bacterial cellulose produced in HS (synthetic) medium. In addition, the produced BCs were characterized by Fourier transform infrared spectroscopy (FT-IR), thermogravimetry (TGA), X-ray diffraction (XRD) and scanning electron microscope (SEM) analyses. This study would extend the list of valuable waste products for BC production that may be used in industry for current and future applications.

## 2. Material and methods

### 2.1. Bacterial strain

In this work, *Komagataeibacter xylinus* (BCRC12334, previously known *Acetobacter xylinum*) gifted by Cheng-Kang Lee (National Taiwan University of Science and Technology) was used for bacterial cellulose production.

### 2.2. Substrate and characterization

Olive oil mill wastewater (OOMW) obtained from Dizem olive oil factory (Çanakkale, Turkey) was used as substrate. The substrate was sterilized by heating at 121°C for 15 min in an autoclave. Then, it was centrifuged at 4000 rpm for 15 min, and the supernatant was used for the preparation of cultivation media.

The pH level of OOMW was measured using a pH meter (Hanna Instruments, Bedfordshire, UK). The sugar levels (glucose, fructose and sucrose) were analyzed using NH_2_ column (250 mm × 4.6 mm, 5 µm, GL Sciences Inc., Tokyo, Japan) in the HPLC system according to Sar et al. [28]. The HPLC analysis was operated at 25°C with 1 mL/min of acetonitrile (60%, v/v) as the eluent. The standards of sugars (5, 10, 20, 40 and 50 g/L) were used for the calibration curves.

### 2.3. Media preparation and cultivation

To prepare the cultivation media, at the first step, the sugar-free HS medium (5 g/L yeast extract, 5 g/L peptone, 2.7 g/L Na_2_HPO_4_, 1.5 g/L citric acid; pH 4.5 [29]) supplemented with different OOMW fractions (25, 50, 75, 100%, v/v) were used to determine the nutritional effects of OOMW. About 75% and 100% OOMW fractions giving the high amounts of BCs supplemented with yeast extract (5 g/L), yeast extract (5 g/L) and peptone (5 g/L), or HS components (without sugar) were used to determine the effect of HS components on cellulose production. The BCs from the OOMW and HS medium containing glucose (20 g/L) were compared regarding their yields and characterizations.

The bacterial strain was precultured in 20 mL of HS medium in 100 mL Erlenmeyer flasks at 30°C, at a static condition for 3 days. Then, the 2.5 mL of precultures were transferred into the cultivation media prepared from HS or OOMW. Cultures were grown in 100 mL medium in 500 mL Erlenmeyer flasks and cultured at 30°C, at static conditions for 7 days [10].

### 2.4. Characterization of BC samples

The produced BC pellicles were washed with distilled water and treated with 1% (w/v) NaOH solution at 80°C for 1 h [30]. Then, BC was dried in an oven at 60°C to constant weight [10]. The dried cellulose pellicles were used for determining their characterization (FT-IR, TGA, XRD and SEM).

Dried BC samples were analyzed by using an FT-IR spectrometer (Bio-Rad Tropical Option for FTS 175C) in the range of 4000–600 cm^−1^ [25].

Thermogravimetric analysis (TGA) of BC samples was carried out with a Mettler Toledo thermal analysis system, TGA/SDTA 851, at a heating rate of 10°C/min in an argon flow between 25°C and 900°C [10]. Differential scanning calorimetry (DSC) analysis was used to measure the thermal stability behavior of BC samples. The analysis was performed by using a differential scanning calorimeter (DSC 821e) equipped with Mettler Toledo Star software. The argon flow rate was 50 mL/min. The temperature range was −50°C to 600°C.

XRD patterns were recorded at room temperature using Ni-filtered Kα Cu X-ray radiation (λ = 1.5406 Å). The operating voltage and current were 40kV and 40 mA, respectively. Data were collected in reflection mode in the 10–40, 2θ-range with a step of 0.02° 2θ intervals [10,11]. Percentage of crystallinity (CI %) of BC samples is determined by Equation (1) [31].

(1) $$CI=\frac{Ima-Iam}{Ima}x100$$

where I_ma_ is the maximum intensity of the lattice peak and I_am_ is the intensity diffraction at 2θ = 18°.

BC samples were imaged using a Scanning Electron Microscope (SEM, Phillips XL30 SFEG, the Netherlands) to investigate their micromorphology [11].

### 2.5. Statistical analysis

The software Minitab17® was used for the statistical analysis of the obtained results with ANOVA (analysis of variance). Pairwise comparisons among groups of data were also carried out using the Tukey test. Significant differences were considered at *p*-value <0.05 within a 95% confidence interval. All experiments were performed in triplicate.

## 3. Results and discussion

In this study, the use of OOMW as a potential substrate for BC production was evaluated. For this, the effects of different ratios of OOMW fractions (25–100%) and some additional nutrients (yeast extract, yeast extract and peptone, or HS components, separately) were investigated. Characterizations (FT-IR, TGA, XRD and SEM) of BCs from OOMW were analyzed comparatively with BC produced from synthetic medium HS. The potential uses of BCs produced from OOMW were also evaluated.

### 3.1. Olive oil mill wastewater (OOMW)

The chemical characteristics of OOMW are given in Table 1. The pH of OOMW is slightly acidic (pH = 5.17) and has been similarly reported ranging from 4.14 to 5.7 [32]. OOMW also contained nutrients such as sugars (glucose, fructose and sucrose), organic acids and ethanol [17], which are utilized by bacterial cellulose producer microorganisms [33].

**Table 1.** General characteristics (pH; glucose, g/L; fructose, g/L; sucrose, g/L) of olive oil mill wastewater (OOMW)

Parameters	Contents
pH	5.17
Glucose (g/L)	7.15 ± 0.18
Fructose (g/L)	1.18 ± 0.11
Sucrose (g/L)	3.03 ± 0.24

### 3.2. Bacterial cellulose production

**Figure 1.**
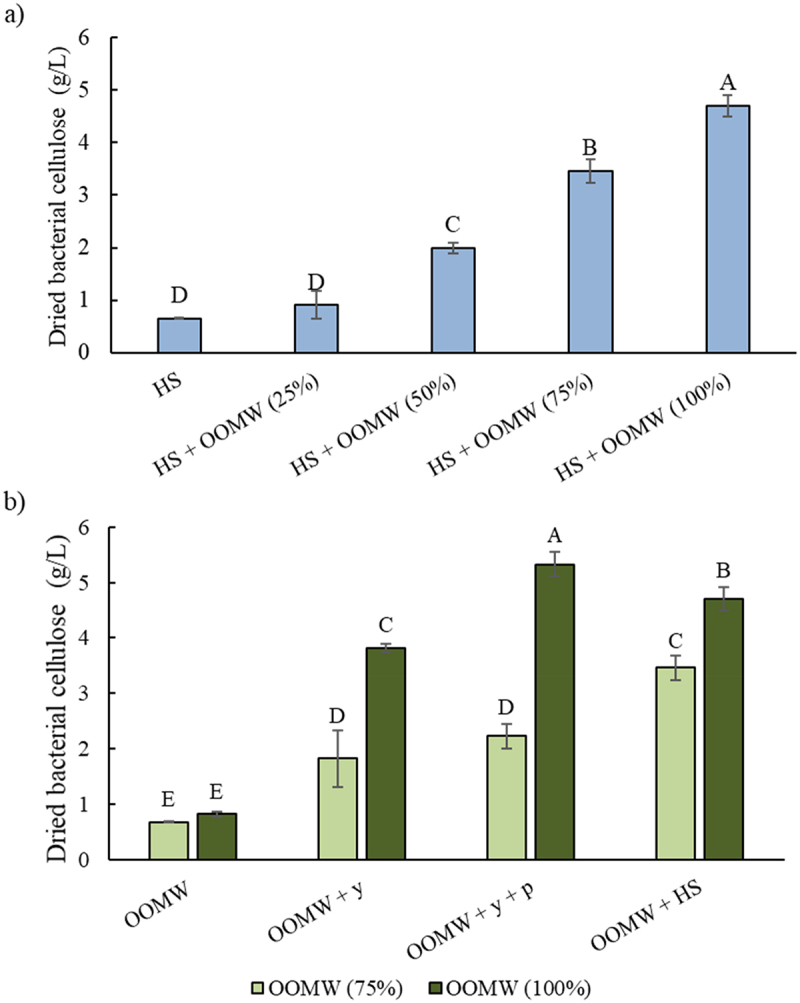
Dried weight of BCs produced in a) HS medium and its enriched with different OOMW fractions (25–100%) and b) OOMW (75% and 100%) media enriched with various nutrients (yeast extract (5 g/L), yeast extract (5 g/L) and peptone (5 g/L), and HS components (without glucose)). Error bars indicate sample standard deviations. Different letters indicate significant differences (P < 0.05).

To determine the usability of olive oil mill wastewater as a substrate for BC production, different ratios of OOMW fractions (25–100%; Figure 1a) were used instead of glucose (2%) in HS medium. In a previous study, Zakaria and Nazeri [34] suggested the optimum pH value of the pineapple waste media for BC production as 5.15. Since the pH value of the OOMW used in this study is 5.17, it was preferred to be used directly without any pH adjustment.

The addition of glucose or OOMW (25%) to the HS medium did not affect BC production (*p* = 0.12). It was determined that the addition of increasing amount of OOMW (from 25% to 100%) to HS medium also increased BC production. This showed that the OOMW did not have any inhibition effect on BC production. Moreover, BC reached 4.70 g/L when undiluted OOMW was used as a supplement (*p* = 0.001). The addition of 75% of OOMW also gave similar yield (4.60 g dried cellulose/liters of added OOMW) with undiluted OOMW supplementation. BC production capacities from OOMW were also evaluated at these dilutions with or without supplements (Figure 1b).

Cellulose production in OOMW media prepared only with 75% or 100% fractions without any supplementation was similar to that of HS media containing glucose (Figure 1). The addition of yeast extract improved the BC production in 100% OOMW medium. When peptone with yeast extract were supplemented, BC productions were increased by 5.5-fold compared to OOMW (100%) without any supplements (*p* = 0.008). The addition of other components of the HS medium (citric acid and sodium phosphate) had an increasing effect in the 75% of OOMW, while it did negatively affect the BC production in 100% OOMW medium. The fact that yeast extract and peptone additions did not have much enhancing effect in the medium containing 75% OOMW that may probably be due to the C/N balance. Since OOMW is nitrogen-poor substrate, external nitrogen sources can be added at different levels. The effects of different nitrogen sources such as corn steep liquor, diammonium phosphate, and ammonium sulfate can be evaluated on BC productions from OOMW [25,35]. In addition, BC productions can be studied by mixing the OOMW with nitrogen-rich wastes.

Glucose and sucrose gave higher yields when they were used as carbon sources for cellulose productions [36]. Besides the carbon and nitrogen sources, ethanol and acetic acid also improved the cellulose productions [37,38]. It can be thought that these carbon sources with other supplements are also effective for productions of BCs from OOMW.

Bacterial cellulose production was affected by some conditions such as media components, temperature, time and pH [39]. It has been reported that fruit juice industry wastes (such as pineapple, citrus, apple) are potential substrates in BC production [39–41]. Additionally, Gomez et al. [25] reported that *Gluconacetobacter sacchari* produced 0.81–0.85 g/L cellulose in dry olive mill residue, similar to the values obtained from OOMW in this study. Additionally, this study showed that OOMW from olive oil industry with different fractions and media supplementation can be a potential substrate to produce BC with high yield.

### 3.3. Characterization of bacterial cellulose obtained from OOMW

**Figure 2.**
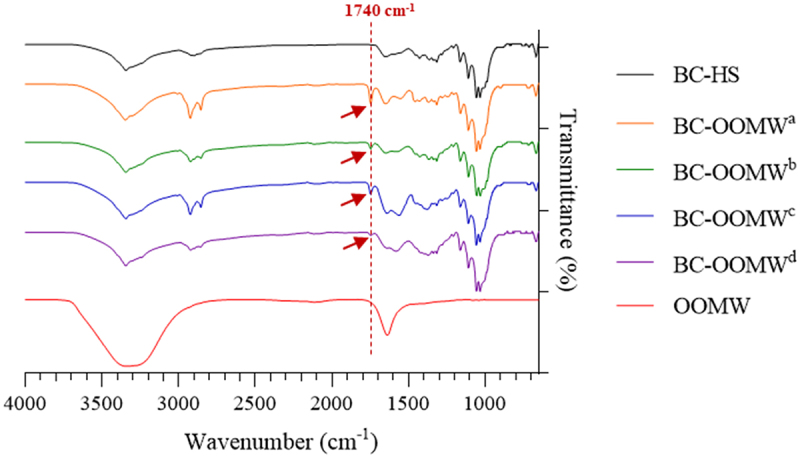
FT-IR analyses of OOMW and BCs produced in HS and OOMW media enriched with various nutrients (OOMW^a^; without supplementation, OOMW^b^; yeast extract (5 g/L), OOMW^c^; yeast extract (5 g/L) and peptone (5 g/L), and OOMW^d^; HS components (without glucose)).

The FT-IR spectrum of both OOMW and BC samples is presented in Figure 2. Co-adsorption for OOMW and BC samples occurred at 3400 cm^−1^ due to H bonded OH groups of alcohol, phenol or organic acids, and H-bonded N-H groups [42,43]. The strong band of OOMW at 1650 cm^−1^ is characteristic of aromatic C = C vibrations [43]. The BC membranes presented typical cellulose FT-IR spectra with strong bands around 3300, 2880 and 1100 cm^−1^ in relation to vibrations of the O–H, C–H, and C–O–C groups of cellulose, respectively [44,45]. The band at 1740 cm^−1^ seen in cellulose samples produced in OOMW is associated with C = O, which does not contain carboxylic acids and aldehydes [42]. Interestingly, this band was detected in BCs produced from OOMW, but not in BC produced from glucose (Figure 2). The band at 1740 cm^−1^ may be related to the fatty acids [46], and this can be interpreted as tyrosol and hydroxytyrosol [47] naturally found in OOMW may be absorbed into the cellulose samples, although it was not clearly observed in only OOMW itself.

**Figure 3.**
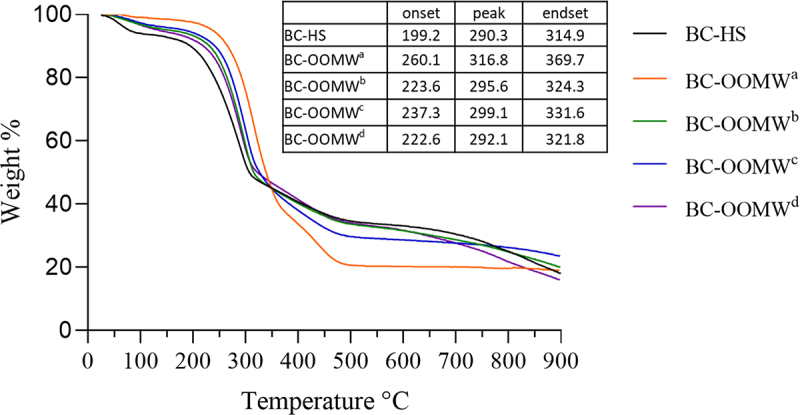
TGA curves of BC samples produced in HS and OOMW media enriched with various nutrients (OOMW^a^; without supplementation, OOMW^b^; yeast extract (5 g/L), OOMW^c^; yeast extract (5 g/L) and peptone (5 g/L), and OOMW^d^; HS components (without glucose)).

The TGA curves of BC samples are shown in Figure 3. Approximately 5% weight loss between 25°C and 120°C may be related to the moisture absorbed by the BC samples (Table 2). Although the weight loss of cellulose samples was stable up to 200°C, it was determined that there was an increase in the reduction in weight loss of cellulose (except BC-OOMW) after this temperature. Similarly, the maximum thermal decomposition temperatures were determined as 316.8°C and 290.3°C in BCs produced from OOMW and glucose, respectively. Thermal degradation behavior may vary depending on the characterization of the cellulose samples such as molecular weight, crystallinity, and fiber orientation [44,48]. A rapid reduction in the weight of the BC-OOMW from this maximum temperature was determined. Then, more than 60% of BC samples were decomposed at 550°C. The weight loss of BC samples was consistent with the findings of previous results [48–50].

**Table 2.** The residual amount (%) of BCs, produced from different media (HS and OOMW), based on the TGA analysis

Temperature (°C)	Weight of BCs (%)				
	BC-HS	BC-OOMW^a^	BC-OOMW^b^	BC-OOMW^c^	BC-OOMW^d^
25	100.0	100.0	100.0	100.0	100.0
50	98.2	100.5	99.4	99.6	99.5
100	94.0	99.1	96.8	97.4	96.7
150	92.8	98.5	95.2	95.9	94.5
200	89.4	97.6	93.3	94.3	92.0
250	76.7	93.3	85.6	88.3	83.6
300	52.7	75.8	58.5	63.4	57.4
350	45.1	44.7	45.0	44.9	46.7
400	40.7	33.7	40.2	38.0	41.5
450	37.0	25.1	36.3	32.5	36.7
500	34.6	20.7	33.7	29.7	34.1
550	33.8	20.4	32.7	29.1	33.0
600	33.1	20.3	31.6	28.7	31.7
650	32.1	20.2	30.2	28.2	29.9
700	30.5	20.1	28.7	27.7	27.7
750	28.2	20.0	27.1	27.1	25.1
800	25.0	19.6	24.9	26.3	21.8
850	21.4	19.5	22.5	25.1	18.8
900	18.0	19.0	20.0	23.5	15.9

^a^without supplementation, ^b^yeast (5 g/L) supplementation, ^c^yeast (5 g/L) and peptone (5 g/L) supplementation, ^d^HS medium components supplementation (without glucose) described in Material Methods

**Figure 4.**
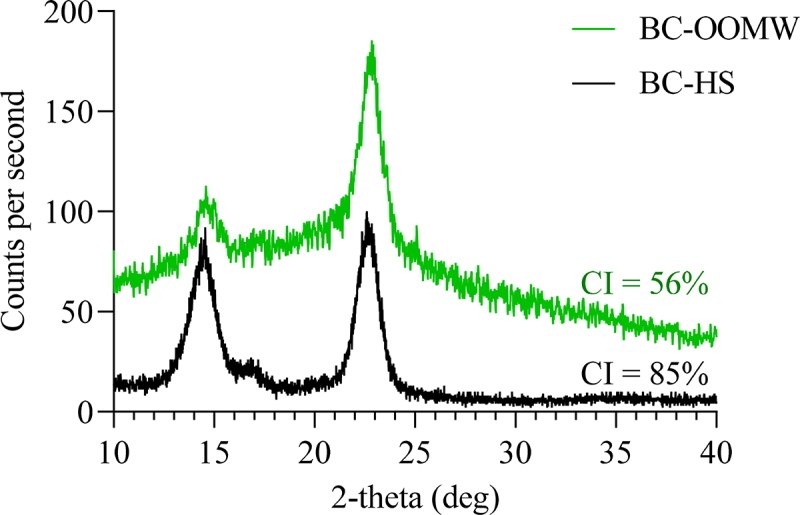
The XRD patterns of BCs produced in HS and OOMW media.

The XRD patterns of BCs produced in HS and OOMW (100% with yeast and peptone) media are shown in Figure 4. The patterns (2θ = 14.4°, 16.4° and 22.7°) are similar for both BCs. The use of OOMW instead of glucose in HS medium reduced the crystallinity index of BC. Similar results have also been reported for BC production when molasses has been used [51–53]. This is probably due to the reduction of hydrogen bonding between fibrils, resulting in a decrease in both the degree of polymerization and the degree of crystallinity [54]. BCs with a lower crystallinity index have a better water absorption ability [55].

**Figure 5.**
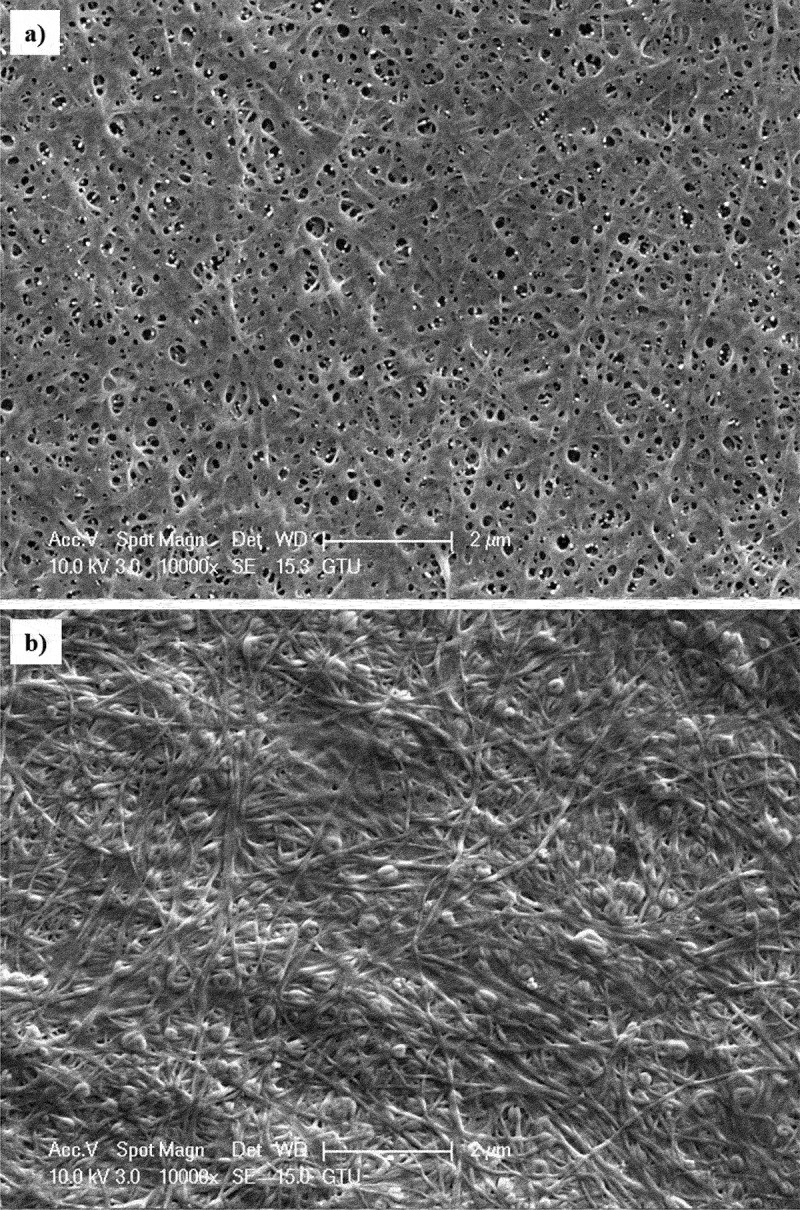
SEM images of BCs produced in a) HS and b) OOMW media.

The SEM images of BC samples are shown in Figure 5. SEM images showed that the BC samples contained an extraordinary cellulose nanofibril network. It was determined that cellulose produced from glucose has clearly porous structures, while cellulose produced from OOMW lacks visible porous networks.

### 3.4. Potential applications of bacterial cellulose

BC can be used in various industries such as food, biomedical, cosmetics, textile and electronics [56]. BC’s most popular product is ‘*nata-de-coco*’, a dessert in gel form, in the South-East Asia [57]. It needs to be determined that it is suitable for food because of the ingredients from the OOMW. In addition, BC from OOMW can be a good candidate biomaterial for cosmetic and biomedical purposes [8,9,12,56] because OOMW contains natural bioactive compounds [23,47]. Alternatively, since it is a natural carbon source, it can be used in electrical/electrochemical applications [10,11]. Behera et al. [58] also reported that Kombucha fermentation is economically feasible with a reasonable rate of return, with the advantage of potential uses of BC. In addition, the use of various substrates in BC production, such as OOMW, will also contribute to waste management.

**Figure 6.**
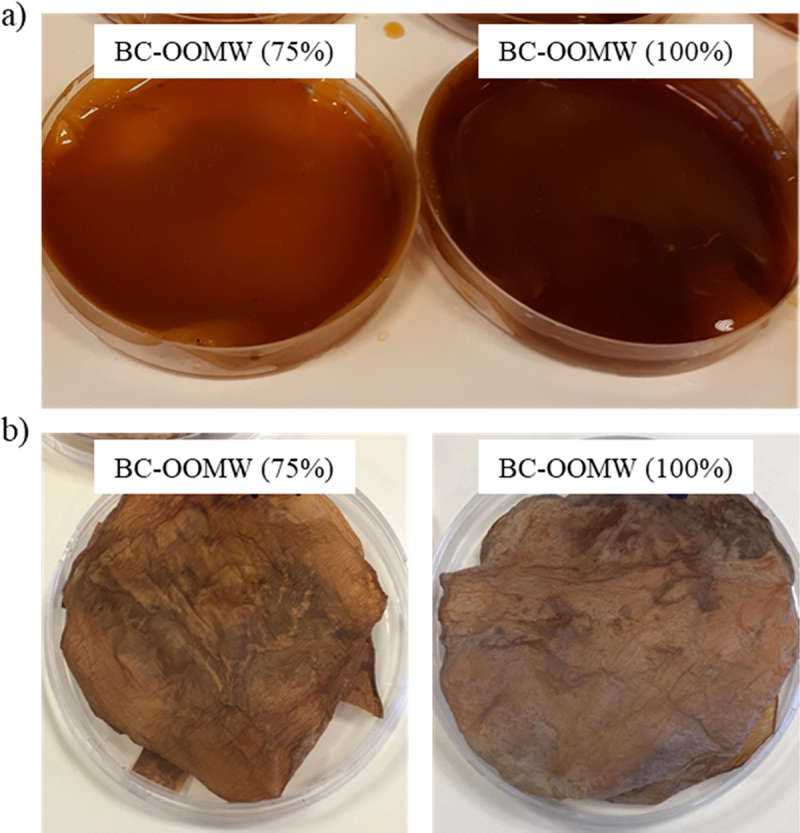
BC pellicles obtained from OOMW media a) wet form and b) dry form.

BC pellicles from OOMW have been dark in color (Figure 6). Producing cellulose in dark color will also allow natural coloring of *nata*. Similarly, co-culture fermentation of the *nata* producer bacteria with natural pigment producer *Monascus purpureus* gave the coloring of *nata*, which can be a new-generation vegetarian food [59,60]. Moreover, dark-colored BC also presents an image like red meat (Figure 6a). On the other hand, dried BC materials (Figure 6b) have similar appearance to brown leather and old paper textures. These colored BCs can be an attractive biomaterials/biofabrics in the packaging, paper and textile industries.

## 4. Conclusion and future perspectives

The present study indicated that OOMW released from olive oil process can be used as nutrient and carbon sources for BC production. Although BC produced in the OOMW medium gave similar BC yields with reference medium HS, OOMW needs additional nitrogen sources due to its nitrogen deficiency. Since the addition of yeast extract and peptone increased BC production, different and inexpensive nitrogen sources can be evaluated in future studies. In addition, the BCs from OOMW media had different characteristics compared to BC from HS containing glucose. Therefore, large-scale productions of BCs from OOMW to determine its potential use for different purposes such as textile and food packing material should be further investigated.
